# International travel as risk factor for *Chlamydia trachomatis* infections among young heterosexuals attending a sexual health clinic in Melbourne, Australia, 2007 to 2017

**DOI:** 10.2807/1560-7917.ES.2019.24.44.1900219

**Published:** 2019-10-31

**Authors:** Ei T Aung, Eric PF Chow, Christopher K Fairley, Jane S Hocking, Catriona S Bradshaw, Deborah A Williamson, Marcus Y Chen

**Affiliations:** 1 Melbourne Sexual Health Centre, Alfred Health, Melbourne, Australia; 2 Central Clinical School, Faculty of Medicine, Nursing and Health Sciences, Monash University, Melbourne, Australia; 3 School of Population and Global Health, University of Melbourne, Melbourne, Australia; 4 Microbiological Diagnostic Unit Public Health Laboratory, Department of Microbiology and Immunology, The University of Melbourne at The Doherty Institute for Infection and Immunity, Melbourne, Australia

**Keywords:** chlamydia, risk factors for chlamydia, travellers, sexually transmitted infections, STIs, sexually transmitted diseases

## Abstract

**Background:**

International travel is considered a risk factor for acquiring *Chlamydia trachomatis*; however, there are little empirical data to support this.

**Aim:**

To examine the prevalence and risk factors for *Chlamydia trachomatis* infections among heterosexual international travellers (n = 28,786) attending the Melbourne Sexual Health Centre (MSHC), Australia, compared to Australian residents (n = 20,614).

**Methods:**

We conducted a repeated cross-sectional study and analysed sexual behaviours and chlamydia positivity among heterosexual males and females aged ≤ 30 attending MSHC for the first time between January 2007 and February 2017. ‘Travellers’ were defined as individuals born outside of Australia who had resided in the country < 2 years. Associations between patient characteristics and chlamydia positivity were examined.

**Results:**

Chlamydia positivity was higher among travellers (11.2%) compared with Australian residents (8.5%; p < 0.001). Male travellers had higher chlamydia positivity (12.1%) than Australian males (9.3%; p < 0.001), as did female travellers (10.4%) compared with Australian females (7.7%; p < 0.001). Travellers had a higher mean number of sexual partners than Australian residents among males (5.7 vs 4.7; p < 0.001) and females (3.6 vs 3.2; p < 0.001). Travellers from the United Kingdom, Europe, Ireland and New Zealand accounted for 29.6%, 21%, 8.5% and 5.8% of *C.* *trachomatis* infections, respectively. Chlamydia in males and females was associated with younger age (≤ 25), inconsistent condom use, a higher number of sexual partners (≥ 4 partners) and being a traveller (p < 0.001).

**Conclusions:**

We found that international travel is an independent risk factor for chlamydia among young heterosexual travellers in Australia, who should therefore be a target group for chlamydia prevention.

## Introduction

*Chlamydia trachomatis* infection is one of the most common bacterial sexually transmitted infections (STIs) worldwide, with an estimated 131 million global cases in 2012 [1]. While most genital chlamydial infections are asymptomatic in both males and females, they are the main cause of non-gonococcal urethritis in males [2]. In females, chlamydia is a known cause of pelvic inflammatory disease and complications such as infertility [3]. Newman et al. estimated the pooled global prevalence of chlamydia among those 15–49 years to be 4.2% among females and 2.7% among males, with regional variations [4]. Travellers may be at particular risk of acquiring STIs such as chlamydia, as well as transmitting STIs to local populations [5,6].

A number of studies have found travel to be associated with increased high-risk sexual behaviours such as condomless sex, multiple sex partners, casual sex partners and sex with partners from countries with higher STI prevalence than one’s home country [6-11]. To date, however, there are few studies that have compared the prevalence of STIs between travellers and local residents, or that have assessed travel as an independent risk factor for STI acquisition [12,13]. A previous study showed that chlamydia prevalence was 1.4 times higher among backpackers attending a sexual health clinic in Sydney compared to local residents [7]. However, other studies comparing chlamydia prevalence in backpackers and local residents in Australia and the United Kingdom (UK) had differing results, with some studies showing no significant differences in prevalence [13-15]. This could be partly due to limited sample sizes in these studies. Therefore, larger studies are required to assess the association between travel and STI acquisition.

The aim of this study was to identify risk factors for *C. trachomatis* infections and compare chlamydia positivity between young heterosexual international travellers and young heterosexual Australian residents attending the Melbourne Sexual Health Centre (MSHC, the only major public STI service in Victoria, Australia) using a large, retrospective dataset. Travellers to Australia do not typically have access to Medicare (Australia’s government-funded universal healthcare), unless they are from a country that has a reciprocal healthcare agreement with Australia, and therefore need to pay for medical services; however, patients attending MSHC are not required to have Medicare and are treated free of charge. MSHC operates as a walk-in clinic, whereby patients are assessed and prioritised based on the presence of symptoms suggestive of an STI, and risk factors for acquiring an STI, and are offered testing and treatment of STIs. Therefore, it is imperative to understand the risk factors associated with travel to provide appropriate and inclusive service to the travellers. It is also important to know if travellers are a high-risk population that does require testing and prioritising to justify the high expenditure associated with STI testing and treatment. Our hypotheses are that *C. trachomatis* infections are higher in travellers and that travel is an independent risk factor for chlamydia.

## Methods

### Study design and population

This was a repeated cross-sectional study of heterosexual men and women aged ≤ 30 years who attended MSHC between 1 January 2007 and 28 February 2017. During the study period, patients who presented to the MSHC with a higher sexual risk or reported genital symptoms suggestive of an STI, e.g. urethral discharge or dysuria in males and vaginal discharge in females, or who requested asymptomatic STI screening were offered STI screening. Patients were assessed first (brief history and/or limited examination) by nurses to allocate them in order of priority for consultation with a clinician. Patients reporting sexual contact with a partner diagnosed with chlamydia were recorded as ‘contact with chlamydia’ in the MSHC’s database.

Prior to STI testing, patients completed a series of questions regarding their recent sexual behaviours using computer-assisted self-interview (CASI), including how many sexual partners they had in the last 3 months and 12 months, if condoms were used during vaginal sex and if they had sex with a partner from outside Australia in the last 12 months. Patients were informed that data were routinely collected and de-identified data might be used in research. At the time of the study, the web-based partner notification service *Let Them Know* (https://letthemknow.org.au/) was operational and Health Department contact tracers were rarely involved in partner notification for chlamydia; however, we did not capture how contacts were informed. Only a patient’s first visit and data related to this consultation were included in this study.

### Case definition for travellers and Australian residents

As most travellers attending MSHC held working holiday visas, allowing them to stay in Australia for up to 2 years, in this study ‘travellers’ were defined as individuals born outside of Australia who had resided in Australia for less than 2 years. As it can take up to 5 years to obtain Australian residency, ‘Australian residents’ were defined as individuals who were born in Australia or, if born overseas, who had resided in Australia for more than 5 years.

### Microbiological sample collection

Specimens obtained for chlamydia testing in women included a cervical or vaginal swab or first pass urine, while men provided first pass urine or urethral swab. Women reporting pelvic pain received bimanual examination; diagnosis of pelvic inflammatory disease was made on clinical grounds and was recorded in the clinic’s database. *C. trachomatis* was diagnosed by nucleic acid amplification test (NAAT) using Aptima Combo-2 (Hologic, Marlborough, Massachusetts, United States (US)) from March 2015 and, prior to that, BD ProbeTec ET (Becton Dickinson, Maryland, US) amplified DNA Assays using strand displacement amplification. *C. trachomatis* infections were defined as those testing positive by either method.

Data on demographic characteristics and sexual behaviour variables were extracted, together with data on chlamydia testing and results data, for all new patients aged ≤ 30 years who attended the MSHC during the study period. This study only included individuals aged ≤ 30 years because Australian guidelines recommend opportunistic screening of sexually active males and females in this age group, as it has the highest prevalence of *C. trachomatis* [14]. Patients were excluded from the analysis if they reported sex with same-sex individuals, transgender individuals or sex workers.

Chlamydia positivity, expressed as a percentage, was calculated as the number of positive cases divided by the number of chlamydia tests. It was compared between Australian residents and travellers.

### Statistical analysis

Statistical analyses were performed using SPSS version 23 (IBM, Chicago, US). Chlamydia positivity included positive test results from asymptomatic patients that were screened for chlamydia, as well as those who reported genital symptoms before being tested for chlamydia. Wilson’s confidence interval (CI) for proportion was used to calculate CI. A chi-squared test was used to compare chlamydia positivity between different subgroups, categorised according to chlamydia risk factors. These subgroups included: (i) contacts with *C. trachomatis* infection, (ii) age, (iii) number of sex partners in the last 12 months, (iv) condom use, (v) presentation with genital symptoms, (vi) sex with overseas partners in the last 12 months and (vii) traveller status. Univariable and multivariable logistic regression were performed to examine factors associated with chlamydia positivity. Factors were also compared between travellers and residents. Factors with p < 0.05 in the univariable analysis were included in the multivariable analysis. A chi-squared trend test was used to examine the annual trend of chlamydia positivity.

### Ethical approval

Ethical approval for the study was obtained from the Alfred Hospital Research Ethics Committee, Melbourne, Australia (number 541/17).

## Results

### Demographic characteristics

During the study period, between 1 January 2007 and 28 February 2017, there were 55,840 clinical consultations for heterosexual patients aged ≤ 30 years; of these, 6,440 (11.5%) were excluded, as no chlamydia testing was performed. Of the 49,400 patients included in the analysis, 28,786 were travellers (12,657 men and 16,129 women) and 20,614 were Australian residents (10,913 men and 9,701 women). The median age was 24 years (interquartile range (IQR): 22–27 years) in both travellers and Australian residents. Travellers had a higher mean number of sexual partners in the last 12 months than Australian residents, among both males (5.7 vs 4.7; p < 0.001) and females (3.6 vs 3.2; p < 0.001).

### Chlamydia positivity

Chlamydia positivity among travellers and residents is shown in Table 1. The chlamydia positivity was higher among travellers 11.2% (3,218/28,786; 95% CI: 10.8–11.6), compared with Australian residents 8.5% (1,762/20,614; 95% CI: 8.2–8.9; p < 0.001). Among males, travellers had higher chlamydia positivity than Australian residents (12.1% vs 9.3%; p < 0.001). This applied to both men with genital symptoms (16.1% vs 12.6%; p < 0.001) and asymptomatic men (10.2% vs 7.5%; p < 0.001). Similarly, chlamydia positivity among females was higher in travellers compared with Australian residents (10.4% vs 7.7; p < 0.001). This also applied to females with genital symptoms (10.0% vs 6.8%; p < 0.001) and asymptomatic females (11.1% vs 8.6%; p < 0.001).

**Table 1:** Comparison of chlamydia positivity between international travellers and Australian residents, Melbourne, Australia, 2007–2017 (n = 49,400)

Characteristics	Travellers (n = 28,786)			Residents (n = 20,614)			p value
	Tested positive for chlamydia	Tested for chlamydia	Positivity % (95% CI)	Tested positive for chlamydia	Tested for chlamydia	Positivity % (95% CI)	
**Males**							
All males	1,537	12,657	12.1 (11.6–12.7)	1,020	10,913	9.3 (8.8–9.9)	< 0.001
Genital symptoms	667	4,154	16.1 (15.0–17.2)	499	3,957	12.6 (11.6–13.7)	< 0.001
No genital symptoms	870	8,503	10.2 (9.6–10.9%)	521	6,956	7.5 (6.9–8.1)	< 0.001
Reported contact with chlamydia	414	1,537	26.9 (24.8–29.2)	271	1,020	26.6 (24.0–29.4)	0.90
**Females**							
All females	1,681	16,129	10.4 (10.0–10.9)	742	9,701	7.7 (7.1–8.2)	< 0.001
Genital symptoms	704	7,062	10.0 (9.3–10.7)	295	4,348	6.8 (6.1–7.6)	< 0.001
No genital symptoms	1,028	9,234	11.1 (10.5–11.8)	463	5,416	8.6 (7.8–9.3)	< 0.001
Reported contact with chlamydia	317	805	39.4 (36.1–42.8)	162	342	47.4 (42.1–52.7)	0.01

CI: confidence interval.

**Figure 1:**
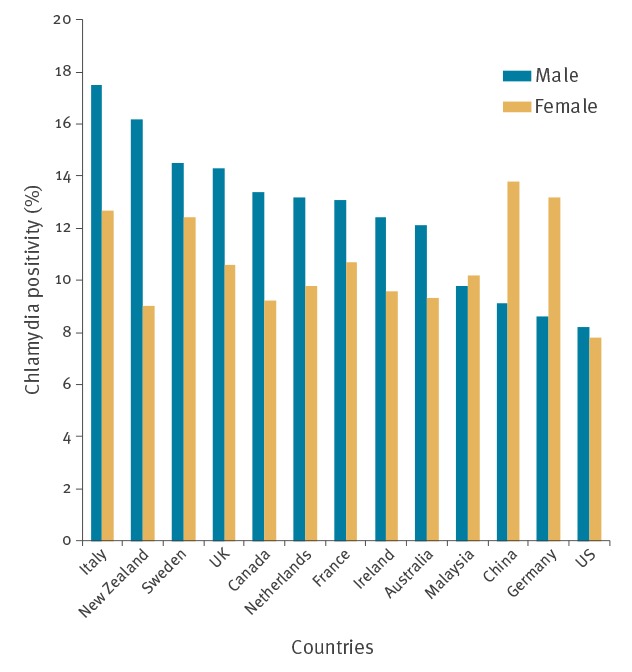
Comparison of chlamydia positivity between males and females, by country of birth, Melbourne, Australia, 2007–2017

UK: United Kingdom; US: United States.

Chlamydia positivity among travellers, by sex and country of origin, are shown in Table 2 and Figure 1. The largest group of travellers was from the UK, which accounted for 26.3% of all travellers in the study. Chlamydia positivity among travellers from the UK was 14.3% (95% CI: 13.2–15.5) for males and 10.6% (95% CI: 9.7–11.6) for females. The travellers who made up the next largest group were from European Union (EU) countries other than the UK, which accounted for 23% of travellers. Chlamydia positivity among EU travellers, excluding UK travellers, was 13.5% (95% CI: 12.3–14.7) in males and 10.8% (95% CI: 10.0–11.8) in females.

**Table 2:** Chlamydia positivity and proportion of total chlamydia cases among travellers, by country of origin, Melbourne, Australia, 2007–2017 (n = 49,400)

Country	Males^a^ (n = 23,570)			Females^b^ (n = 25,830)		
	Tested positive for chlamydia	Tested for chlamydia	Chlamydia positivity % (95% CI)	Tested positive for chlamydia	Tested for chlamydia	Chlamydia positivity % (95% CI)
**European Union countries**						
France	77	586	13.1 (10.6–16.1)	83	779	10.7 (8.7–13.0)
Germany	30	349	8.6 (6.1–12.1)	67	508	13.2 (10.5–16.4)
Ireland	158	1,270	12.4 (10.7–14.4)	109	1,196	9.6 (7.6–10.9)
Italy	47	268	17.5 (13.5–22.5)	28	220	12.7 (9.0–17.8)
Netherlands	23	174	13.2 (9–19.1)	31	316	9.8 (7.0–13.6)
Sweden	25	172	14.5 (10.0–20.6)	95	765	12.4 (10.3–15.0)
UK	518	3,615	14.3 (13.2–15.5)	435	4,117	10.6 (9.7–11.6)
**North America**						
Canada	36	269	13.4 (9.8–18.0)	77	838	9.2 (7.4–11.3)
US	33	403	8.2 (5.9–11.3)	67	864	7.8 (6.2–9.7)
**Asia Pacific**						
China	51	558	9.1 (7.1–11.8)	98	711	13.8 (11.4–16.5)
Malaysia	17	174	9.8 (6.2–15.1)	25	245	10.2 (7.0–14.6)
New Zealand	114	704	16.2 (13.7–19.1)	72	798	9.0 (7.2–11.0)
**Other countries^d^**	**609**	**5,995**	**10.2 (9.4–11.0)**	**777**	**7,689**	**10.1 (9.5–10.1)**

UK: United Kingdom; US: United States.

^a^ Total chlamydia cases among male travellers: 1,537.

^b^ Total chlamydia cases among female travellers: 1,681.

^d^ The 12 countries accounting for the greatest number of chlamydia cases are listed. The remaining cases of chlamydia among travellers are grouped under ‘Other countries’, which includes 179 countries. In total, 191 countries are included.

The 12 countries accounting for the greatest number of chlamydia cases are listed. Chlamydia positivity of Australian residents was included for comparison.

**Figure 2:**
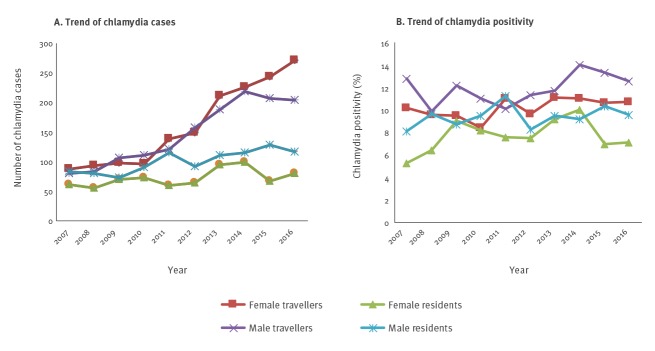
Number of chlamydia infections and chlamydia positivity among international travellers and Australian residents, by sex, Melbourne, Australia, 2007–2016

The number of *C. trachomatis* infections diagnosed in travellers and residents during the study period, by year, is shown in Figure 2A. Over the course of the study period, there was a 2.5-fold increase in the number of infections diagnosed among male travellers at the clinic and a 3-fold increase among female travellers, while the number remained relatively steady among male and female residents. Trends in chlamydia positivity over time in travellers and residents, by sex, are shown in Figure 2B. Overall, there was no significant increase in chlamydia positivity among travellers or residents during the study period, with the exception of chlamydia positivity among male travellers, which showed a significant upward trend (p = 0.01). Among chlamydia-positive women, pelvic inflammatory disease was diagnosed in 4.8% of the travellers (80/1,681; 95% CI: 3.8–5.9; p = 0.3) and 5.9% of the residents (44/742; 95% CI: 4.5–7.9; p = 0.3).

Chlamydia positivity was determined by dividing the number of chlamydia-positive cases by the total number of chlamydia tests. It is expressed as a percentage.

### Risk factors for chlamydia

Potential risk factors were compared between travellers and residents, with males and females combined in each group. Travellers were more likely to have ≥ 4 partners in the last 12 months compared with Australian residents (49.0% (14,099/28,786) vs 42.4% (8,736/20,614); p < 0.001), as well as sexual partners from overseas in the last 12 months (62.8% (7,570/12,050) vs 34.2% (3,547/10,368); p < 0.001). There were no significant differences between travellers and residents, respectively (data not shown), for the following potential risk factors: age groups (for example, 62.9% (18,118/28,786) vs 63.7% (13,141/20,614); p = 0.07, for those aged ≤ 25), consistent condom use in the last 12 months (15.5% (4,207/27,174) vs 15.2% (2,915/19,176); p = 0.4) or contact with chlamydia (6.3% (1,805/28,786) vs 6.3% (1,289/20,6114); p = 0.9).

### Multivariate logistic regression analysis

Associations between potential risk factors and chlamydia positivity among all males and all females (travellers and residents combined) were assessed by multivariate analysis, as shown in Table 3.

**Table 3:** Associations between potential risk factors and chlamydia positivity among all heterosexual males and females aged ≤ 30 years, Melbourne, Australia, 2007–2017(n = 49,400)

Characteristics	Males (n = 23,570)^a^			Females (n = 25,830) ^a^		
	No. of individuals	OR (95% CI, p value)	aOR (95% CI, p value)	No. of individuals	OR (95% CI, p value)	aOR (95% CI, p value)
**Chlamydia contact**						
Yes	1,785	6.6 (6.0–7.4, p < 0.001)	9.4 (8.3–10.6, p < 0.001)	1,309	6.7 (5.9–7.6, p < 0.001)	7.0 (6.2–8.0, p < 0.001)
No	21,785	1 (ref)	1 (ref)	24,521	1 (ref)	1 (ref)
**Age (years)**						
≤ 20	2,028	1.2 (1.0–1.4, p = 0.04)	1.3 (1.1–1.5, p = 0.008)	3,603	1.9 (1.7–2.2, p < 0.001)	2.0 (1.7–2.3, p < 0.001)
21–25	11,765	1.4 (1.3–1.6, p < 0.001)	1.4 (1.3–1.5., p < 0.001)	13,863	1.5 (1.4–1.7, p < 0.001)	1.5 (1.3–1.6, p < 0.001)
26–30	9,777	1 (ref)	1 (ref)	8,364	1 (ref)	1 (ref)
**Number of partners in last 12 months**						
≤ 3	11,063	1 (ref)	1 (ref)	15,502	1 (ref)	1 (ref)
≥ 4	12,507	1.7 (1.6–1.9, p < 0.001)	1.6 (1.4–1.7, p < 0.001)	10,328	1.6 (1.5–1.7, p < 0.001)	1.4 (1.3–1.5, p < 0.001)
**Condom use in last 12 months**						
Inconsistent	18,506	3.2 (2.7–3.8, p < 0.001)	2.7 (2.3–3.2, p < 0.001)	20,722	2.1 (1.8–2.5, p < 0.001)	2.0 (1.7–2.3, p < 0.001)
Always or non-penetrative sex	3,721	1 (ref)	1 (ref)	3,401	1 (ref)	1 (ref)
**Genital symptoms**						
Yes	8,111	1.7 (1.6–1.8, p < 0.001)	2.8 (2.5–3.1, p < 0.001)	11,410	0.9 (0.8–1.0, p = 0.002)	1.1 (1.0–1.3, p = 0.004)
No	15,459	1 (ref)	1 (ref)	14,420	1 (ref)	1 (ref)
**Sex overseas in last 12 months**						
Yes	11,117	1.2 (1.1–1.3, p < 0.001)	1.1 (1.0–1.2, p = 0.3)	11,624	1.3 (1.2–1.4, p < 0.001)	1.1 (1.0–1.2, p = 0.02)
No	11,301	1 (ref)	1 (ref)	13,021	1 (ref)	1 (ref)
**Traveller**						
Yes	12,657	1.3 (1.2–1.5, p < 0.001)	1.4(1.2–1.5, p < 0.001)	16,129	1.4 (1.3–1.5, p < 0.001)	1.4 (1.3–1.6, p < 0.001)
No	10,913	1 (ref)	1 (ref)	9,701	1 (ref)	1 (ref)

aOR: adjusted odds ratio; CI: confidence interval; OR: odds ratio; ref: reference.

^a^ There are missing data for characteristics such as condom use in last 12 months (1,707 female and 1,343 males) and sex overseas in last 12 months (1,185 females and 1,152 males). The total may not add up due to incomplete computer-assisted self-interviews.

aOR is adjusted for (i) being a sexual contact of person with chlamydia infection, (ii) age, (iii) number of partners in the last 12 months, (iv) condom use in the last 12 months, (v) presence of genital symptoms suggestive of sexually transmitted infection, (vi) having had sexual partners from overseas in the last 12 months and (vii) being a traveller.

Among males, chlamydia positivity was independently associated with: (i) reporting sexual contact with a partner with chlamydia (adjusted odds ratio (aOR): 9.4; 95% CI: 8.3–10.6), (ii) younger age (aOR: 1.3; 95% CI: 1.1–1.5 for those aged ≤ 20 ), (iii) ≥ 4 female partners in the last 12 months (aOR: 1.6; 95% CI: 1.4–1.7), (iv) inconsistent condom use in the last 12 months (aOR: 2.7; 95% CI: 2.3–3.2), (v) genital symptoms (aOR: 2.8; 95% CI: 2.5–3.1) and (vi) being a traveller (aOR: 1.4; 95% CI: 1.2–1.5).

Among females, chlamydia positivity was independently associated with: (i) reporting sexual contact with a partner with chlamydia (aOR: 7.0; 95% CI: 6.2–8.0), (ii) younger age (aOR: 2.0; 95% CI: 1.7–2.3 for those aged ≤ 20), (iii) ≥ 4 male partners in the last 12 months (aOR: 1.4; 95% CI: 1.3–1.5), (iv) inconsistent condom use in the last 12 months (aOR: 2.0; 95% CI: 1.7–2.4), (v) genital symptoms (aOR: 1.1; 95% CI: 1.0–1.3) and (vi) being a traveller (aOR: 1.4; 95% CI: 1.3–1.6).

Sex with a partner from overseas in the last 12 months was significantly associated with chlamydia infection in females (aOR: 1.1; 95% CI: 1.0–1.2; p = 0.02), but not in males (aOR: 1.1; 95% CI: 1.0–1.2; p = 0.10).

Potential risk factors for *C. trachomatis* infection and their association with chlamydia positivity among travellers and residents are shown in Table 4.

**Table 4:** Association of risk factors for chlamydia infection among travellers and Australian residents, Melbourne, Australia, 2007–2017 (n = 49,400)

Characteristics	Travellers^a^ (n = 28,786)			Australian residents^a^ (n = 20,614)		
	No. of individuals	OR (95% CI, p value)	aOR (95% CI, p value)	No. of individuals	OR (95% CI, p value)	aOR (95%CI)
**Chlamydia contact**						
Yes	1,805	6.7 (6.1–7.4, p < 0.001)	7.8 (6.9–8.9, p < 0.001)	1,289	6.9 (6.0–7.8, p < 0.001)	9.3 (7.7–11.1, p < 0.001)
No	26,981	1 (ref)	1 (ref)	19,325	1 (ref)	1 (ref)
**Age (years)**						
≤ 20	2,368	1.5 (1.3–1.8, p < 0.001)	1.6 (1.4–2.0, p < 0.001)	3,263	1.7 (1.4–1.9, p < 0.001)	1.5 (1.3–1.9, p < 0.001)
21–25	15,750	1.5 (1.4–1.6, p < 0.001)	1.4(1.4–1.7, p < 0.001)	9,878	1.4 (1.2 -1.6, p < 0.001)	1.4 (1.2–1.6, p < 0.001)
26–30	10,668	1 (ref)	1 (ref)	7,474	1 (ref)	1 (ref)
**Number of partners in last 12 months**						
≤ 3	14,687	1 (ref)	1 (ref)	11,878	1 (ref)	1 (ref)
≥ 4	14,099	1.6 (1.5–1.7, p < 0.001)	1.5 (1.3–1.6, < 0.001)	8,736	1.7 (1.6–1.9, p < 0.001)	1.6 (1.4–1.8, p < 0.001)
**Condom use in last 12 months**						
Inconsistent	22,967	2.6 (2.2–3.0, p < 0.001)	2.3 (2.0–2.8, < 0.001)	16,261	2.7 (2.2–3.3, p < 0.001)	2.4 (1.9–3.1, p < 0.001)
Always or non-penetrative sex	4,207	1 (ref)	1 (ref)	2,915	1 (ref)	1 (ref)
**Genital symptoms**						
Yes	11,216	1.2 (1.1–1.3, p < 0.001)	1.9 (1.7–2.1, < 0.001)	8,305	1.2 (1.1–1.4, p < 0.001)	2.6 (2.3–3.0, p < 0.001)
No	17,570	1 (ref)	1 (ref)	12,309	1 (ref)	1 (ref)
**Sex overseas in last 12 months**						
Yes	7,570	1.2 (1.1–1.3, p = 0.0.004)	1.0 (0.9–1.2, p = 0.6)	3,547	1.0 (0.9–1.2, p = 0.6)	1.1 (0.9–1.2, p = 0.4)
No	4,480	1 (ref)	1 (ref)	6,821	1 (ref)	1 (ref)
**Sex**						
Male	12,657	1.2 (1.1–1.3, p < 0.001)	1.2 (1.0–1.3, p = 0.005)	10,913	1.2 (1.2–1.4, p < 0.001)	1.2 (1.0–1.5, p = 0.06)
Female	16,129	1 (ref)	1 (ref)	9,701	1 (ref)	1 (ref)

aOR: adjusted odds ratio; CI: confidence interval; OR: crude odds ratio; ref: reference.

^a^ There are missing data for characteristics such as condom use in last 12 months (1,612 travellers and 1,418 residents) and sex overseas in last 12 months (16,736 travellers and 10,246 residents). The total may not add up due to incomplete computer-assisted self-interview.

aOR is adjusted for (i) being a sexual contact of person with chlamydia infection, (ii) age, (iii) number of partners in the last 12 months, (iv) condom use in the last 12 months, (v) presence of genital symptoms suggestive of sexually transmitted infection, (vi) having had sexual partners from overseas in the last 12 months and (vii) sex.

## Discussion

To our knowledge, this is the largest study of chlamydia among international travellers, with over 3,000 travellers diagnosed with chlamydia over a 10-year period. Chlamydia positivity was significantly higher among heterosexual male and female travellers attending the MSHC between 2007 and 2017 compared with Australian residents; travel was an independent risk factor for *C. trachomatis* infection among both males and females, justifying maintaining ongoing free access to healthcare for travellers attending our service.

We found that younger age (≤25 years), inconsistent condom use and a higher number of sexual partners during the last 12 months were risk factors for chlamydia, findings that are consistent with other studies [16-18]. Over the study period, between 2007 and 2017, the number of chlamydia infections diagnosed among travellers attending MSHC increased, overtaking the number of diagnoses among Australian residents in the same age group. These results suggest that young international travellers visiting Australia should be a target group for chlamydia prevention and screening strategies. It is notable that 4.8% of female travellers with chlamydia were also diagnosed with pelvic inflammatory disease, a serious complication of *C. trachomatis* infection in females. Therefore, screening and prompt treatment of *C. trachomatis* infection should be encouraged to prevent morbidity arising from the infection among female travellers.

Consistent with previous studies, we also found that young, heterosexual international travellers were more likely to engage in higher risk sexual behaviours, such as having a high number of partners [8-10,12,15,19,20]. These studies have suggested that more casual sexual relationships during travel may be a result of physical separation from regular partners in an individual’s country of origin and removal of social factors that might inhibit such sexual behaviours [6,8,13,19-21]. Higher alcohol consumption, increased sexual activity with multiple partners, and partners from countries with higher prevalence of STIs have also been identified as risk behaviours for chlamydia among travellers [7,10,15,18,21]. It has been estimated that the risk of developing an STI increases up to 3-fold in people who engage in casual sex [8], with previous studies reporting the prevalence of travel-associated casual sex ranging between 20% and 35%, with the prevalence of condomless sex between 17% and 49% [8,19].

In our study, being a traveller was associated with chlamydia infection, independent of sexual risk factors such as having a higher number of partners or inconsistent condom use; this suggests that travellers are exposed to additional risk factors for chlamydia acquisition. One hypothesis is that travellers in our study had sex with other travellers (i.e. sexual network factors) and, therefore, were more likely to have sex with individuals who also have a higher likelihood of being infected with chlamydia [22]. One of the factors most strongly associated with chlamydia in both travellers and residents was sex with a partner who reported having chlamydia. MSHC did not routinely record whether sexual partners were travellers or not; therefore, we were not able to examine this.

### Limitations

There are several limitations to this study. As it was conducted at a single clinic (MSHC), the findings may not be generalisable to international travellers around the world. We were only able to investigate prevalence of chlamydia, as screening for other STIs, e.g. gonorrhoea, is not routinely recommended among heterosexuals in Australia according to national guidelines [23]. Patients with chlamydia infection in the study included asymptomatic individuals and those presenting with chlamydia-associated symptoms. Patients that were high risk for chlamydia infection or those presenting with genital symptoms were more likely to attend MSHC than patients requesting anonymous screening; this may have resulted in a higher number of chlamydia infections reported in this study. Furthermore, we did not examine changes in sexual behaviour over time, which could influence changes in positivity rates for chlamydia.

The number of international visitors to Australia increased 1.6-fold over the study period, from 5 million in 2007 to 8 million in 2017; approximately 50% of these visitors were in Australia for holidays [24]. It is not known to what extent the travellers in this study acquired chlamydia infections in their home countries or during travel to other countries, as opposed to in Australia. Therefore, this could have resulted in a higher number of *C. trachomatis* infections among travellers.

### Conclusions

Our study suggests that young, heterosexual travellers are at risk of chlamydia**infection and would benefit from pre-travel advice aimed at reducing the risk of STIs, including safe sex education and promotion of condom use. This could be administered in primary care settings, travel clinics or sexual health clinics, with follow-up STI screening at post-travel consultations. Sexually active travellers should be encouraged to attend STI screening and should seek STI testing if they develop genital symptoms in their destination country. When planning sexual health service delivery, policymakers should consider young, heterosexual international travellers as a high-risk population and should implement strategies to increase international travellers’ access to sexual health services abroad. Future research incorporating epidemiological typing of *C. trachomatis* with data on travel could help define the extent of transmission of chlamydial strains between countries and subsequent dissemination within destination countries.
